# Epidemiological features of SARS-CoV-2 Omicron infection under new control strategy: a cross-sectional study of the outbreak since December 2022 in Sichuan, China

**DOI:** 10.1186/s12889-023-17361-5

**Published:** 2023-12-08

**Authors:** Runyou Liu, Yang Zhang, Jingxuan Ma, Hongjian Wang, Yajia Lan, Xuefeng Tang

**Affiliations:** 1 West China School of Public Health and West China Fourth Hospital, Sichuan University, Chengdu, Sichuan 610041 P.R. China; 2 Sichuan Center for Disease Control and Prevention, Chengdu, Sichuan 610041 P.R. China; 3 Department of Periodical Press and National Clinical Research Center for Geriatrics, West China Hospital, Sichuan University, Chengdu, Sichuan 610041 P.R. China; 4 West China Hospital, Chinese Evidence-Based Medicine Center, Sichuan University, Chengdu, Sichuan 610041 P.R. China

**Keywords:** Epidemiological features, SARS-CoV-2, Outbreak, COVID-19, Omicron

## Abstract

**Background:**

A major shift in the “dynamic zero-COVID” policy was announced by China’s National Health Commission on December 7, 2022, and the subsequent immediate large-scale outbreak of SARS-CoV-2 infections in the entire country has caused worldwide concern. This observational cross-sectional study aimed to describe the epidemiological characteristics of this outbreak in Sichuan, China.

**Methods:**

All data were self-reported online by volunteers. We described the epidemic by characterizing the infection, symptoms, clinical duration, severity, spatiotemporal clustering, and dynamic features of the disease. Prevalence ratio (PR), Odds ratios (ORs) and adjusted ORs were calculated to analyze the associations between risk factors and infection and the associations of risk factors with clinical severity using log-binomial and multivariable logistic regression models; 95% confidence intervals (CIs) and Wald test results were reported. The prevalence rates and clinical severity among different subgroups were compared using the Chi-square and trend Chi-square tests.

**Results:**

Between January 6 and 12, 2023, 138,073 volunteers were enrolled in this survey, and 102,645 were infected with COVID-19, holding a prevalence rate of 74.34%; the proportion of asymptomatic infections was 1.58%. Log-binomial regression revealed that the risk of infection increased among those living in urban areas. Multivariable logistic regression analysis showed that female sex, chronic diseases, older age and the fewer doses of vaccine received were associated with an increased risk of severe clinical outcomes after infection. We estimated the mean reproduction number during this pandemic was 1.83. The highest time-dependent reproduction number was 4.15; this number decreased below 1 after 11 days from December 7, 2022. Temporal trends revealed a single peak curve with a plateau pattern of incidence during the outbreak, whereas spatiotemporal clustering analysis showed that the onset in 21 cities in the Sichuan province had four-wave peaks.

**Conclusions:**

The peak of the first wave of Omicron infection in Sichuan Province had passed and could be considered a snapshot of China under the new control strategy. There were significant increases in the risk of severe clinical outcomes after infection among females, with chronic diseases, and the elderly. The vaccines have been effective in reducing poor clinical outcomes.

## Background

 COVID-19 has been a global epidemic for three years and has infected approximately 760 million people worldwide and killed 6.8 million people as of March 19, 2023, according to the statistics released by the World Health Organization (WHO) [1]. The Omicron variant was first detected in mid-November 2021 in South Africa and was named “variant of concern” by the WHO. Due to its infectivity and immune evasion capacity [2], Omicron has rapidly replaced Delta as the dominant strain worldwide, causing two unprecedented outbreak peaks. The first outbreak occurred in late 2021 and early 2022, mainly in the United States and European countries, whereas the second wave burst occurred in the Western Pacific by the end of 2022, with mainland China as the core region [1]. On January 27, 2023, the International Health Regulations (2005) Emergency Committee regarded the ongoing coronavirus disease pandemic as a public health emergency of international concern owing to its capacity to cause substantial damage to health and health systems [3].

Previous studies conducted during the Omicron wave showed that epidemiological characteristics varied in areas owing to different control policies and pre-infection and vaccination patterns in the population. A study reported that the effective reproduction number was 2.19 in the rapid outbreak of the Omicron variant in South Africa, where fewer than one in ten people were fully vaccinated [4]. The Real-time Assessment of Community Transmission-1 study estimated that the regional doubling time for Omicron in England in December 2021 ranged from 1.6 to 2.5 days [5], and another study showed that living in urban areas was associated with a higher risk of swab positivity [6]. A case-control study in Brazil found that the effectiveness of past infections in preventing reinfection during the Omicron wave was low [7]. Moreover, previous studies have found that the Omicron strain is less virulent than the other strains and mainly causes asymptomatic or mild infection [8, 9]. Early reports have shown that vaccines attenuate COVID-19 symptoms, duration, and viral RNA shedding [10, 11]. However, the epidemiological and clinical characteristics of Omicron in mainland China during the transition period of the strategy from “dynamic zero-COVID” to “coexistence with the virus” are yet to be elucidated.

By the end of 2022, China implemented the “dynamic zero-COVID” policy for nearly three years since the outbreak of COVID-19 [12], and had > 90% of the Chinese population having had two doses of the COVID-19 vaccine [13]. However, the Omicron strain first caused a large local outbreak in Shanghai in April 2022 and continuously caused localized outbreaks in many cities, including Ningbo and Zhuhai [14–16], bringing huge challenges to the current policy. China changed its response strategy for COVID-19 on December 7, 2022, by announcing “10 new measures,” which included home isolation or quarantine for individuals with mild symptoms or those who are asymptomatic and the termination of region-wide mass testing [17]. Consequently, coupled with a large, susceptible population, the pandemic immediately spread across the country. This study launched a population-based dynamic online cross-sectional survey after the implementation of these 10 measures to fill the gap in regular surveillance. The main objectives of this study are as follows. First, the study described COVID-19 by exploring the infection status of different subgroups in communities and identifying the risk factors for infection. Second, we assessed the clinical features of symptomatic cases, including the occurrence of symptoms, duration, and severity, and detected associations between clinical severity and individual characteristics. Third, the study described the epidemic process by characterizing the spatiotemporal and dynamic features to provide effective information to the Department of Public Health.

## Methods

### Study design and participants

Three rounds of online surveys were conducted by the Sichuan provincial and city-level Centers for Disease Control and Prevention (CDC) between December 17, 2022, and January 12, 2023, covering more than 750,000 residents in all 21 cities in Sichuan Province. The questionnaire was carefully optimized according to the results of a previous survey round; therefore, this study draws on data from the third round of surveying. The study was conducted from January 6 to January 12, 2023, and included 19 questions referring to the demographics of participants, infection status, clinical symptoms, and so on. All questions were closed-form items and did not include any personal private information. Ethical approval for the use of the questionnaire for research purposes in Sichuan Province was obtained from the Sichuan CDC Ethics Committee (approval number: SCCDCIRB-No.2023-005). We published questionnaires on the official WeChat public account of the Sichuan CDC and called on staff at all levels in Sichuan province to advertise the survey. Furthermore, some local Internet media outlets, including the Sichuan Daily and Chengdu Publishing also transmitted the survey on their platforms.

All residents of Sichuan Province were recruited as volunteers for the survey, and informed consent was obtained from every participant. The first page of the questionnaire provided an introduction to the survey objective and application of the results. The participants can also proxy-report to their families; therefore, the survey further covers infants, children, and elderly groups who may be out of the reach of our questionnaire.

### Outcomes

The following 13 symptoms were investigated: fever, cough, fatigue, headache, expectoration, sore throat, muscle pain, stuffy and runny nose, loss of taste and smell, chills, diarrhea, vomiting, and bloody stool. COVID-19 infection cases referred to participants who were positive for SARS-CoV-2 nucleic acid by RT-PCR or antigen testing by nasopharynx test paper using respiratory specimens or had not yet accepted coronavirus nucleic acid or antigen testing with one or more of the clinical symptoms mentioned above since December 7, 2022.

Cases of infection combined with one or more symptoms were considered symptomatic. The clinical duration was defined as the symptom duration of symptomatic patients who recovered during the survey period. Symptomatic cases, including recovered and unrecovered patients whose symptoms lasted for ≥ 14 days, were analyzed to evaluate the severity of clinical outcomes. Cases were divided into two types: mild- or medium-type, which comprised patients with fewer than five symptoms lasting < 14 days, and severe type, which comprised patients who had ≥ 5 lasting ≥ 14 days.

### Statistical analysis

Baseline characteristics are presented as numbers (%) for categorical variables. We stratified the infection rates by geographical location, demographics, vaccination, and smoking characteristics (region, sex, age group, underlying conditions, vaccination, and smoking status). We used log-binomial regression model and calculated prevalence ratio (PR) to estimate the association between region and infection. We calculated the odds ratios (ORs) and adjusted ORs for infection of the other characteristics. The adjustment used a multivariable logistic regression model to analyze the associations between risk factors and infection, and 95% confidence intervals (CIs) and Wald test results were reported. In this regression analysis, all stratification characteristics were included as categorical variables. Multivariable ordinary logistic regression was also employed to investigate the odds of clinical severity between mild- or medium-, and severe cases, adjusting for sex, age, underlying conditions (chronic obstructive pulmonary disease [COPD], diabetes, hypertension, cardiac-cerebral vascular disease, nephropathy, other respiratory diseases, autoimmune diseases [AID], and tumors), vaccination status, and smoking status. The Chi-square and trend chi-square tests were used to compare the infection rates and clinical severity of all subgroups.

We calculated the daily incidence of symptomatic cases in different cities in Sichuan Province and implemented a spatiotemporal cluster analysis to explore different waves in various regions. Based on the assumption that the total population during the survey period was relatively stable, we also estimated the mean reproduction number and time-dependent reproduction number in this pandemic by using “R_0_” package [18]. Hierarchical clustering method was used inthe spatiotemporal cluster analysis through “pheatmap” package in R software. The “average” clustering method and “correlation” distance calculation method were applied in the parameter settings [19]. We used R version 4.2.1 (The R Foundation for Statistical Computing, Vienna, Austria) for statistical analysis, and *P*-values < 0.05 were considered statistically significant.

## Results

### Baseline characteristics

**Fig. 1:**
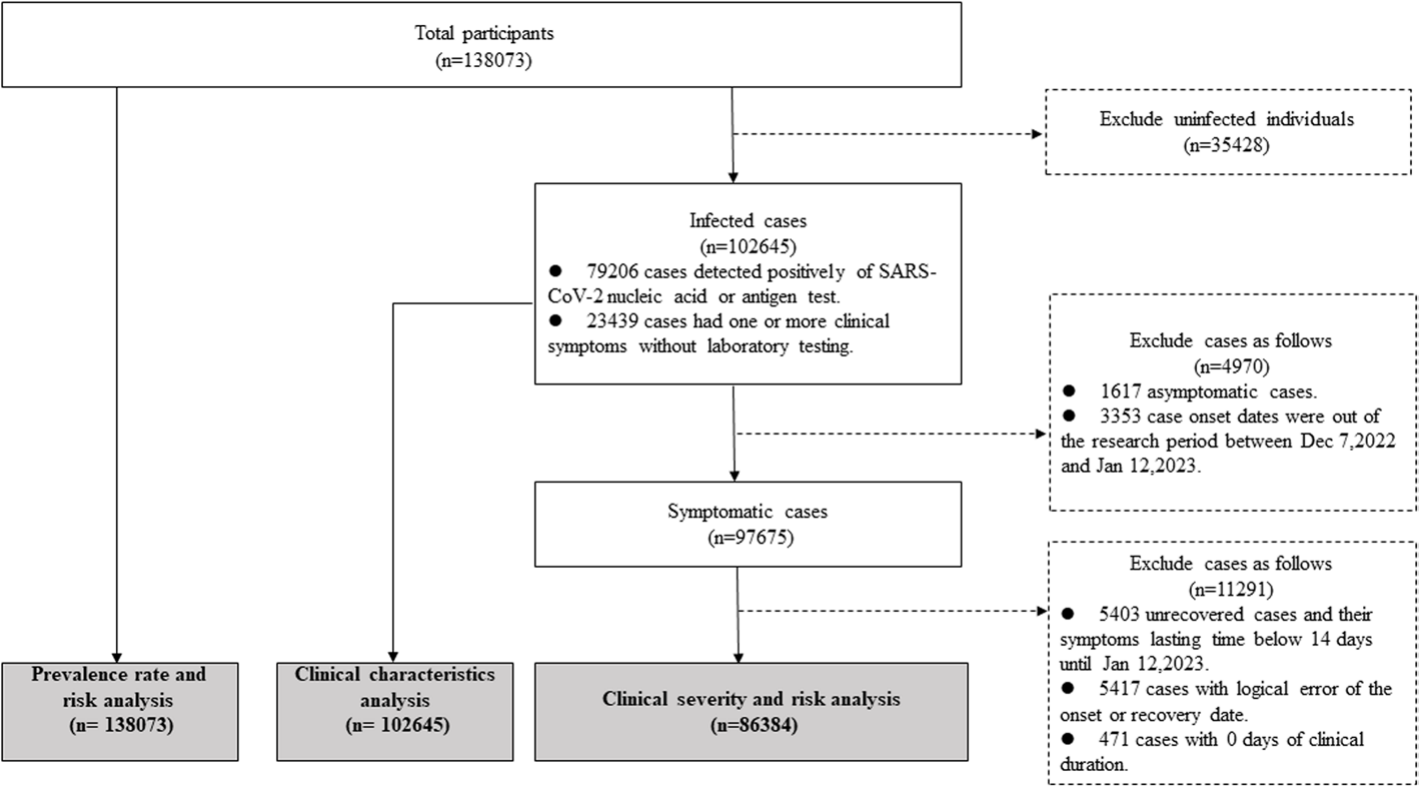
Flow diagram of the participant inclusion and exclusion criteria for defining study cohorts for analysis

 In total, 138,073 participants were enrolled in the survey between January 6 and January 12, 2023. All participants information were collected in the infection risk analysis. However, after applying the exclusion criteria, 97,675 symptomatic cases were included in the spatiotemporal and dynamic features analysis, 86,384 symptomatic cases were included in the symptom and severity analysis, and 58,917 symptomatic cases who had recovered were included in the clinical duration analysis (Fig. 1).

The baseline characteristics stratified by demographic features and vaccination histories are shown in Table 1. Of all participants, 97,810 residents were from urban areas (70.84%), and 40,263 residents were from rural areas (29.16%). More than half of the participants were female (56.95%), and most participants were aged 26–50 years (58.73%). A total of 82.11% of the participants had no chronic diseases before being affected by COVID-19, and most were non-smokers (83.26%). Regarding vaccination history, most participants were administered three vaccine doses (79.1%).

### Prevalence rate and risk factors

Among the 138,073 participants, 102,645 were infected by SARS-CoV-2 Omicron including 79,206 cases (77.16%) detected positively of nucleic acid or antigen test and 23,439 cases (22.84%) had one or more clinical symptoms without laboratory testing, with a prevalence rate of 74.34%. Notably, the prevalence rate among urban residents was 80.09%, 32.67% higher than that among rural residents (60.73%). The prevalence rate in women was 77.72%, 11.24% higher than that in men (69.87%), and the prevalence rate in participants with chronic diseases (77.55%) was slightly higher than the average. Across all age categories, the highest incidence rate was in the 26–50-year-old age group (77.88%), followed by the 51–65-year-old age group (75.28%), whereas the remaining groups had a lower-than-average rate. Surprisingly, smokers (64.94%) had a lower prevalence rate than non-smokers (76.23%). Overall, the prevalence rate was higher in those who had been vaccinated (75.51%) than in unvaccinated populations, but the prevalence rate among those who completed four doses of the vaccine (37.02%) was below average. The differences in the prevalence rates of all the stratified characteristics mentioned above were statistically significant (*P* < 0.001). Table 1 presents the remaining details regarding the participants and infection outcomes.

**Table 1:** Demographic characteristics of participants and prevalence rates

Subgroup	No. of participants	No. of infected cases	The prevalence rates	*p*-value^$^
	(n,%) N1 = 138,073	(n,%) N2 = 102,645	(%, 95%CI)	
Region				
Rural	40,263 (29.16)	24,308 (23.68)	60.37 (59.90 ~ 60.85)	< 0.001
Urban	97,810 (70.84)	78,337 (76.32)	80.09 (79.84 ~ 80.34)	
Sex				
Male	59,443 (43.05)	41,530 (40.46)	69.87 (69.50 ~ 70.23)	< 0.001
Female	78,630(56.95)	61,115 (59.54)	77.72 (77.43 ~ 78.02)	
Age group, years				
0 ~ 6	1368 (0.99)	737 (0.72)	53.87 (51.23 ~ 56.52)	< 0.001
7 ~ 12	4821 (3.49)	1886 (1.84)	39.12 (37.74 ~ 40.50)	
13 ~ 25	18,132 (13.13)	12,395 (12.08)	68.36 (67.68 ~ 69.04)	
26 ~ 50	81,086 (58.73)	63,153 (61.53)	77.88 (77.60 ~ 78.17)	
51 ~ 64	26,449 (19.16)	19,911 (19.4)	75.28 (74.76 ~ 75.80)	
65~	6217 (4.5)	4563 (4.45)	73.40 (72.30 ~ 74.49)	
Underlying conditions^a^				
Without chronic diseases	113,369 (82.11)	83,487 (81.34)	73.64 (73.39 ~ 73.90)	< 0.001
With chronic diseases^b^	24,704 (17.89)	19,158 (18.66)	77.55 (77.03 ~ 78.07)	
Vaccination status				
Unvaccinated	3455 (2.5)	2511 (2.45)	72.68 (71.19 ~ 74.16)	
1 ~ 2does	22,335 (16.18)	15,220 (14.83)	68.14 (67.53 ~ 68.76)	< 0.001
3 does	109,220 (79.1)	83,780 (81.62)	76.71 (76.46 ~ 76.96)	
4 does	3063 (2.22)	1134 (1.1)	37.02 (35.31 ~ 38.73)	
Current smoker				
Yes	23,109 (16.74)	15,008 (14.62)	64.94 (64.33 ~ 65.56)	< 0.001
No	114,964 (83.26)	87,637 (85.38)	76.23 (75.98 ~ 76.48)	

^$^The differences in prevalence rates in different subgroups were analyzed using the Chi-square and trend chi-square tests

^a^The chronic diseases in this study comprised the following eight diseases: chronic obstructive pulmonary disease (COPD), diabetes, hypertension, cardiac-cerebral vascular disease, nephropathy, other respiratory diseases, autoimmune disease (AID), and tumors

^b^“with chronic diseases” indicates that participants suffered from at least one of the chronic diseases and that multiple presenting conditions were possible

**Fig. 2:**
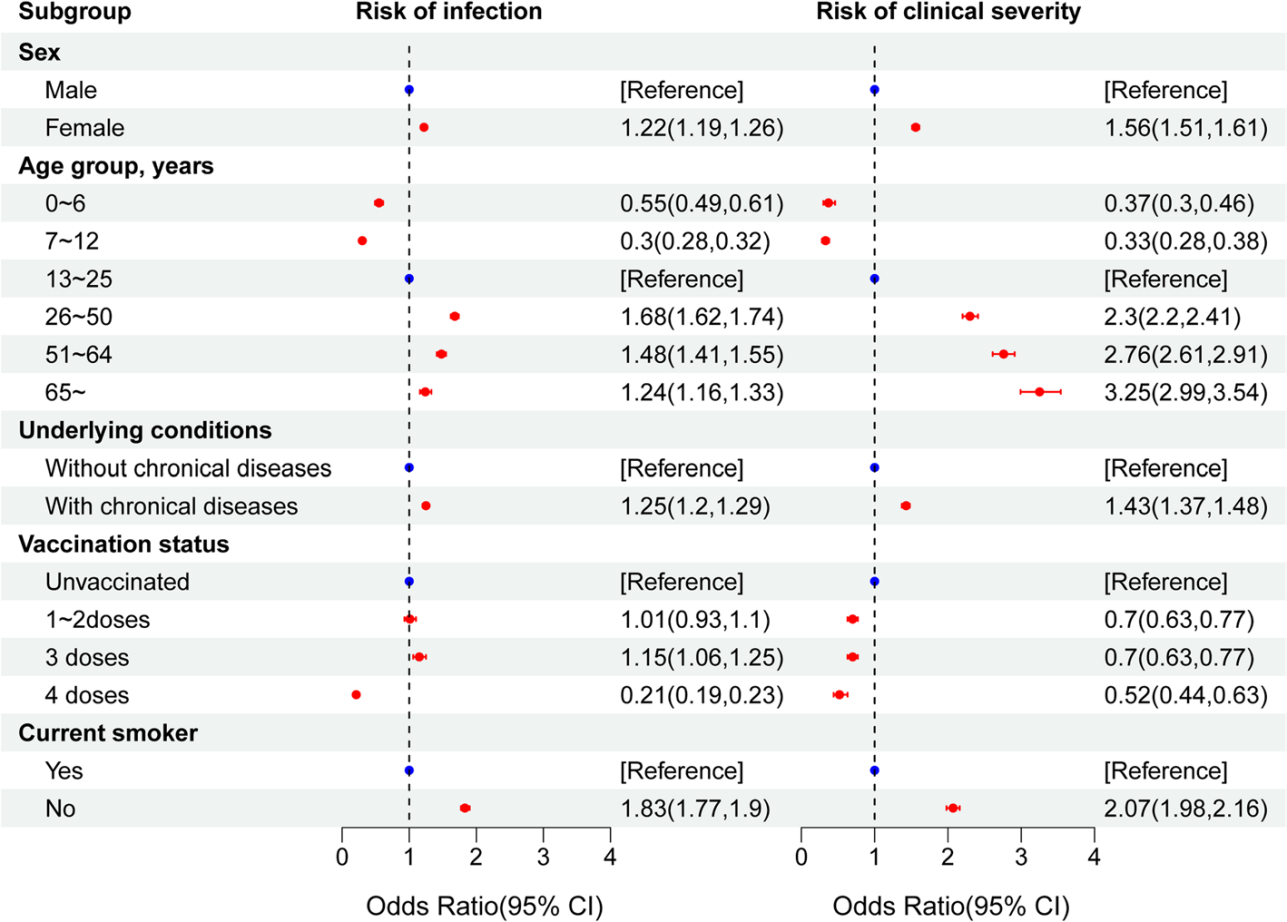
Multivariable logistic regression analysis of SARS-CoV-2 infection and clinical severity factors Multivariate logistic analysis was adjusted for sex, age, underlying conditions, vaccination status, and smoking status. Error bars represent 95% CI. Chronic diseases were indicated as individuals suffering from at least one of the following chronic diseases (multiple presenting conditions were possible): COPD, diabetes, hypertension, cardiac-cerebral vascular disease, nephropathy, other respiratory diseases, AID, and tumors; 8 chronic diseases were included herein

 Log-binomial regression analysis showed that the risk of infection was 1.33 (95% CI, 1.32–1.34) times higher for urban residents than for those in rural areas. The results of the multivariable logistic regression analysis showed that female sex (OR, 1.22; 95% CI, 1.19–1.26), chronic diseases (OR, 1.25; 95% CI, 1.20–1.29), and being a nonsmoker (OR, 1.83; 95% CI, 1.77–1.90) were associated with an increased risk of infection. Furthermore, compared with the 13–25-year-old age group, the 0–6- and 7–12-year-old age groups were associated with a reduced risk of causing infections, while the adjusted risk ORs for increasing infection in the 26–50-year-old, 51–64-year-old, and 65-year-old age groups were 1.68 (95% CI, 1.62–1.74), 1.48 (95% CI, 1.41–1.55), and 1.24 (95% CI, 1.16–1.33), respectively. For individuals who had not been vaccinated, the completion of four doses of vaccination was associated with a reduced risk, whereas the completion of three doses was associated with an increased risk of infection (OR, 1.15; 95% CI, 1.06–1.25). For more details, see Table 1; Fig. 2.

### Clinical manifestation of COVID-19 and risk factors

Of the 102,645 patients in this study, 1617 (1.60%) did not develop any symptoms after infection, and the proportion of asymptomatic infections was 1.58%. By the end of the survey, 58,917 symptomatic cases’ clinical symptoms had completely disappeared, with a recovery rate of 60.32%. The longest clinical duration in recovered cases was 36 days, with a median of 11 days (interquartile range, 7–16 days). For more details, please refer to Table 2. As of January 12, 2023, 27,467 symptomatic cases had not recovered, but their symptom duration was ≥ 14 days. Together with the recovered population, 86,384 symptomatic cases were included in the symptom and severity analysis.

Among the 86,384 patients with clinical symptoms, the mean number of symptoms was 6.21, and the five most common symptoms in terms of incidence were cough (83.74%), fever (77.14%), fatigue (67.37%), muscle pain (62.05%), and sore throat (58.41%). There were 47,116 severe cases and 39,268 light (medium) cases. The incidence of severe disease among female patients was 60.26–31.51% higher than that in males and tended to increase with age (*p* < 0.001). Individuals who had chronic underlying diseases, were not vaccinated, and were non-smokers were more likely to experience severe clinical outcomes, with a prevalence of severe disease of 63.95%, 64.14%, and 57.44%, respectively, in these cases (Table 2).

**Table 2:** Clinical characteristics of symptomatic cases of COVID-19 in Sichuan province

Subgroup	Symptomatic cases (*N* = 86,384)	No. of symptoms (mean, days)	Clinical duration^a^	Clinical Severity		
			(M-IQR, days)	Light/Medium (n,%) (N1 = 39,268)	Severe (n,%) (N2 = 47,116)	*p*-value^$^
Sex						
Male	34,194	6	9 (6 ~ 14)	18,528 (54.18)	15,666 (45.82)	< 0.001
Female	52,190	7	12 (7 ~ 17)	20,740 (39.74)	31,450 (60.26)	
Age group, years						
0 ~ 6	573	6	6 (3 ~ 9)	461 (80.45)	112 (19.55)	< 0.001
7 ~ 12	1328	3	6 (3 ~ 10)	1118 (84.19)	210 (15.81)	
13 ~ 25	9933	4	9 (6 ~ 13)	6293 (63.35)	3640 (36.65)	
26 ~ 50	53,476	6	11 (7 ~ 16)	23,394 (43.75)	30,082 (56.25)	
51 ~ 64	17,127	6	12 (8 ~ 17)	6748 (39.4)	10,379 (60.6)	
65~	3947	6	13 (9 ~ 19)	1254 (31.77)	2693 (68.23)	
Underlying conditions						
Without chronical diseases	68,838	6	10 (7 ~ 15)	32,942 (47.85)	35,896 (52.15)	< 0.001
With chronical diseases	17,546	6	12 (8 ~ 17)	6326 (36.05)	11,220 (63.95)	
Vaccination status						
Unvaccinated	2122	6	12 (7 ~ 17)	761 (35.86)	1361 (64.14)	< 0.001
1 ~ 2does	12,418	6	9 (6 ~ 15)	6389 (51.45)	6029 (48.55)	
3 does	71,073	6	11 (7 ~ 16)	31,721 (44.63)	39,352 (55.37)	
4 does	771	6	10 (6 ~ 14.25)	397 (51.49)	374 (48.51)	
Current smoker						
Yes	11,873	5	8 (5 ~ 13)	7556 (63.64)	4317 (36.36)	< 0.001
No	74,511	6	11 (7 ~ 16)	31,712 (42.56)	42,799 (57.44)	

^$^ Differences in the proportion of different clinical outcomes were analyzed using the Chi-square and trend chi-square tests

^a^Clinical duration refers to symptom duration, which indicates symptomatic cases that have already recovered. However, 58,917 cases were involved in the clinical duration analysis

Multivariable logistic regression analysis showed that female sex (OR, 1.56; 95% CI, 1.51–1.61), having chronic underlying diseases (OR, 1.43; 95% CI, 1.37–1.48), and being a nonsmoker (OR, 2.07; 95% CI, 1.98–2.16) were associated with an increased risk of severe clinical outcomes after infection with COVID-19. Moreover, older age was associated with an increased risk. For example, the risks of severe clinical outcomes in the 26–50-year-old, 51–64-year-old, and 65-year-old age groups were 2.30 times, 2.76 times, and 3.25 times that in the 13–25-year-old age group, respectively. Furthermore, vaccinations were associated with a reduced risk of severe clinical outcomes, and the higher the dose of the vaccine received, the lower the risk of severe clinical outcomes (Table 2; Fig. 2).

### Dynamic features and spatiotemporal clustering analysis

**Fig. 3:**
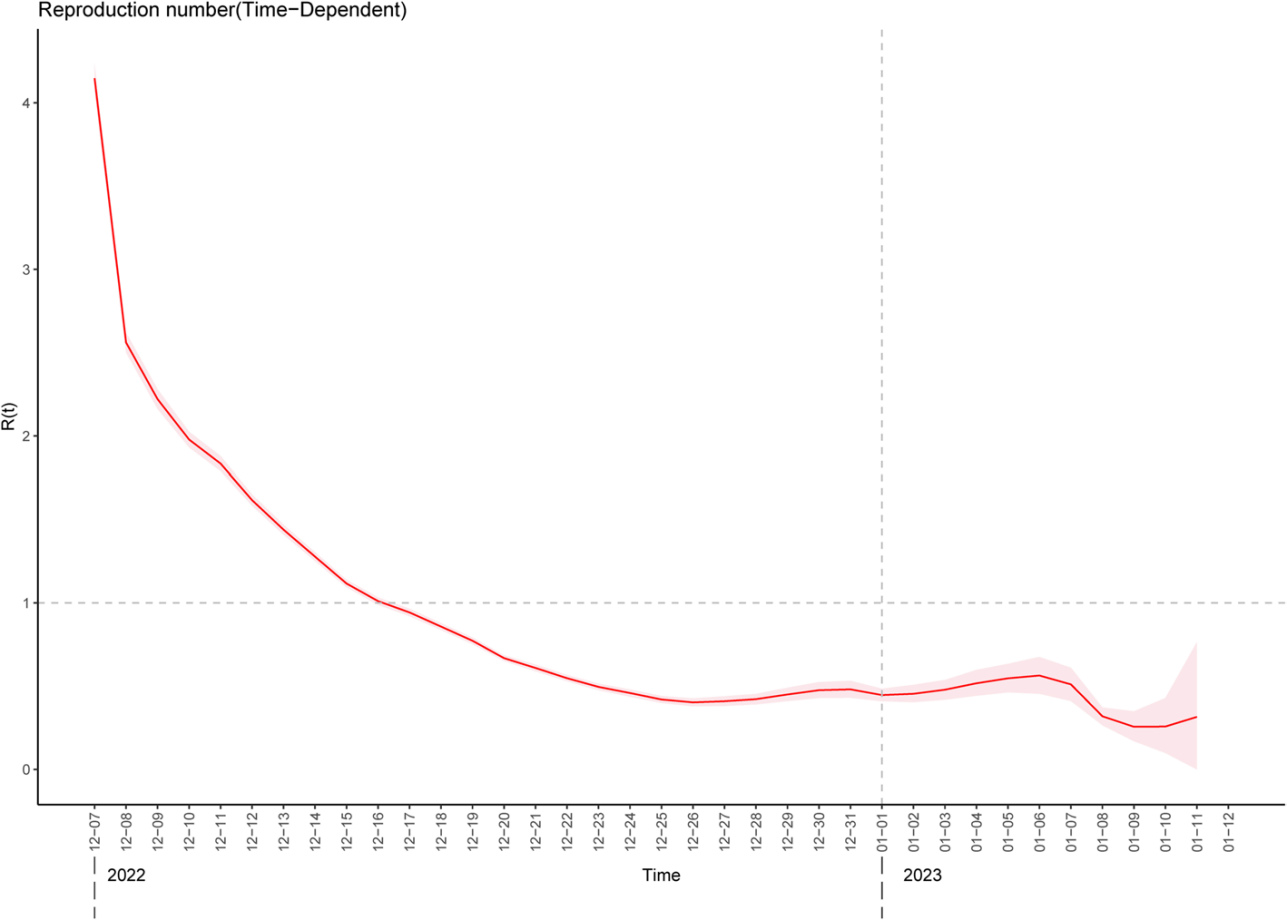
Time-dependent reproduction number trend in Sichuan between December 7, 2022, and January 12, 2023 Assuming a mean Weibull-distributed generation time of 3.56 days and standard deviation of 2.28 days, we estimated the Rt using the “R 0 ” package and daily case incidence data. The red solid line and pink area represent the point estimates and 95% CI, respectively. The horizontal dashed line represents an Rt of 1, and the vertical dashed line represents the start of 2023. Rt, reproduction number

 There were 83.7 million residents in Sichuan Province in 2022. We estimated the mean reproduction number (R) according to the population and prevalence rate, which was 1.8298 (95% CI,1.8295–1.8300). We estimated the daily time-dependent reproduction number (Rt) for the outbreak period in Sichuan province using daily incidence data, assuming a mean Weibull-distributed generation time of 3.56 days and a standard deviation of 2.28 days [20]. Rt was the highest at the beginning of the epidemic, reaching 4.15 (95% CI, 4.06–4.23). Finally, Rt decreased below 1 on December 17, 2022, 11 days after the implementation of the “10 new measures” had been issued (December 7, 2022) (Fig. 3). Moreover, Rt in urban areas (4.10; 95% CI, 4.00–4.19) was lower than that in rural areas (4.51; 95% CI, 4.34–4.68), decreasing below 1 10 days and 14 days after the implementation, respectively.

**Fig. 4:**
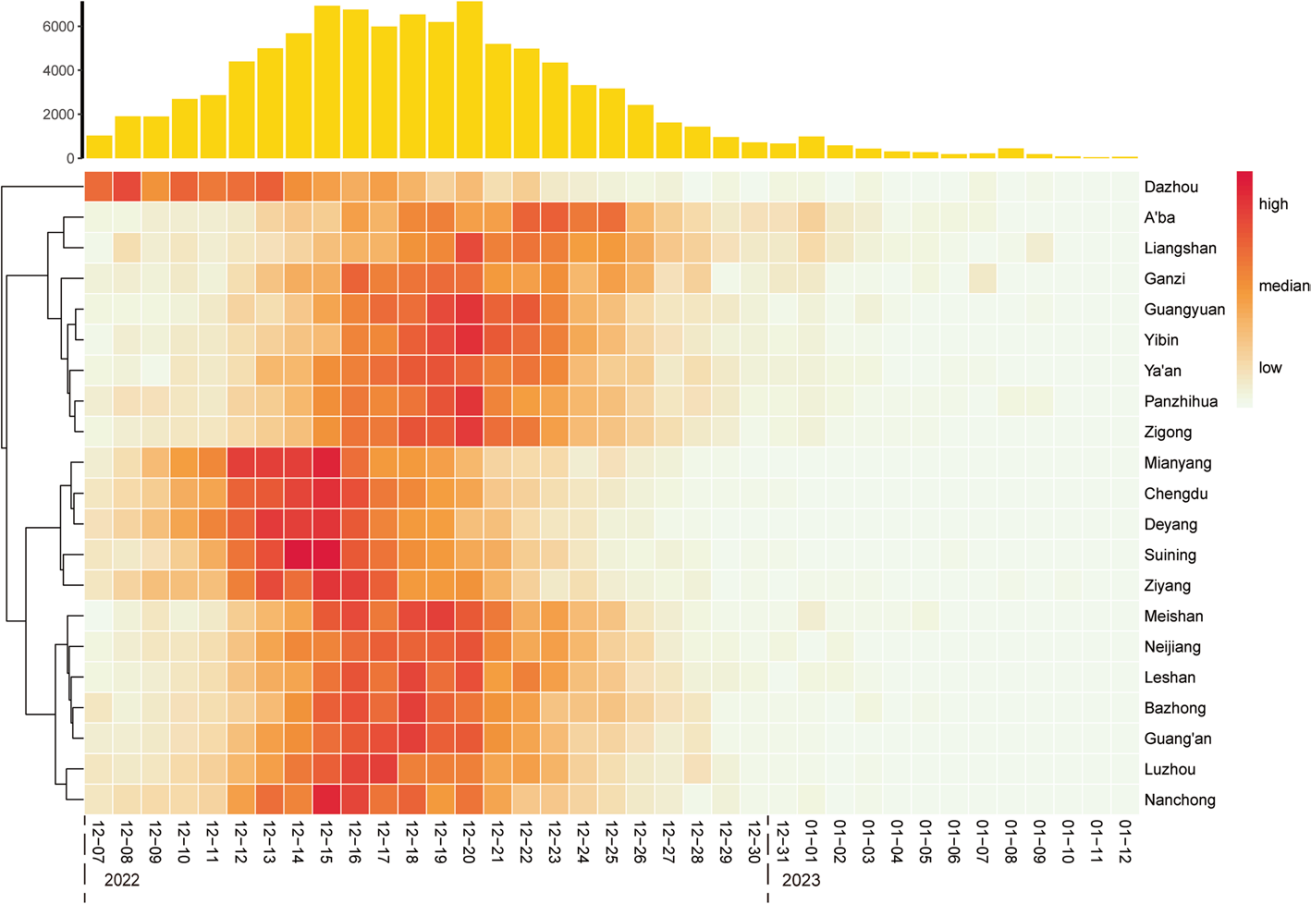
Temporal and Spatiotemporal dynamics of SARS-CoV-2 cases in Sichuan between December 7, 2022, and January 12, 2023 The light-orange bar chart represents the daily incidence of SARS-CoV-2 during this provincial outbreak. We conducted a spatiotemporal clustering analysis of 21 cities in Sichuan Province using daily incidence data from different regions, as shown in the heat map below

 The results of regional distribution showed that the prevalence rate varied greatly from different regions, with more than 50% of the 21 cities in Sichuan province had a rate above 80%. Temporal trends revealed a single peak curve with a plateau pattern of incidence during the outbreak, and the onset of cases was concentrated between December 12 to December 23, 2022. However, the results of the spatiotemporal clustering analysis showed that onset in the 21 cities in Sichuan Province had four-wave peaks. First, onset in Dazhou, eastern Sichuan peaked on December 8. Second, onset in Chengdu, Suining, and the other 5 cities peaked between December 12 and December 15, 2022. The third wave was in Nanchong; onset in the other seven cities peaked between December 15 and 19. The last wave was in Yibin, and onset in the remaining eight cities peaked between December 18 and 20 (Fig. 4).

## Discussion

Since January 20, 2020, COVID-19 has been monitored as a class A communicable disease in China. It requires that all medical institutions and laboratory testing organizations report COVID-19 cases through the National Information System for Disease Control and Prevention within 2 h of detection. However, due to the home isolation of patients with mild or asymptomatic disease and the overloading of medical facilities during the outbreak, we cannot grasp the infection data precisely, which is detrimental to controlling the epidemic. Therefore, we conducted this urgent survey to describe the characteristics of a large-scale outbreak caused by the Omicron variant in Sichuan Province, China after adjusting for the COVID-19 response strategy.

Our Omicron prevalence rate was higher than that of the first wave of Omicron outbreaks in other areas, including South Africa (58% in urban areas by April 2022) [21] and Demark (66% by March 2022) [22], while it was similar to that in Beijing (estimated to be 75.7% on December 22, 2022) and some other major cities (estimated that 60–80% of people were infected) in China [23, 24]. Given that all over the China had implemented a successful “dynamic zero-COVID” strategy before November 2022, our cumulative prevalence rates were extremely low, and population immunity to SARS-CoV-2 was almost entirely vaccine derived when the Omicron wave began. Therefore,our study could be considered a snapshot of China under the new control strategy, although we only had provincial data. It revealed that the maximum effective reproduction number in Sichuan was higher than that in Beijing (4.15 vs. 3.44, respectively) [23], and the mean reproduction number was slightly higher than that in the first wave of the Omicron epidemic in South Korea (1.83 vs. 1.72, respectively) [25]. Nearly 65% of the population was infected in just three weeks, demonstrating that the circulation of the Omicron variant in Sichuan province was more extensive and faster than that in other studies.

The risk of infection among urban residents was 1.33 times higher than that of rural residents; Similarly, in the United Kingdom, the risk of infection among urban residents was 1.24 times that of rural residents [6], which may be related to the high population density and frequent contact among urban populations. Nevertheless, Antonelli et al. suggested that rural areas, especially impoverished areas, are high-risk populations for infection, even if they have been vaccinated [26]. Our province has a large population of migrant workers, which leads to the rural population comprising mainly vulnerable groups, including the elderly and children. Moreover, rural areas may have less robust medical capabilities; therefore, we should pay more attention to the Omicron epidemic in these regions and reasonably allocate medical and health resources to reduce the burden of COVID-19 in rural areas [27].

This study found that the rate of asymptomatic infections among infected residents was only 1.58%, which is much lower than that in Zhuhai City in Guangdong Province (76%) [16] and Ningbo City in Zhejiang Province (54.1%) [28]. The main reason for this was that the cases were at different stages of disease development when they were detected (i.e., under different response strategies for COVID-19). When the “dynamic zero-COVID” policy was being implemented, mass testing had been applied in response to an outbreak and for disease surveillance among populations at risk, aiming to find people with active infection who are asymptomatic or pre-symptomatic so that early quarantine, tracing, and testing of close contacts can interrupt the spread of COVID-19 [29, 30]. Therefore, the sensitivity of surveillance is high, resulting in a high proportion of asymptomatic infections. Moreover, according to the outcomes of this study, most participants were deemed positive for COVID-19 based on the presence of symptoms, which is also an important cause of the low proportion of asymptomatic infections reported in this study.

Cough, fever, and fatigue were the most common clinical symptoms among symptomatic cases, which differs from the initial results of studies conducted in South Africa, the United Kingdom, and other countries [31–33]. Their reports also listed runny nose and headache as the most common symptoms, which may be related to the different characteristics of the Omicron substrain, including ethnicity, previous COVID-19 infection, and vaccination status of the population [7]. The mean number of symptoms was very similar to the findings of Thompson et al. [34], but the clinical duration herein was shorter than that reported by them (12.3 d).

Similar to many previous studies [35–37], we found that chronic diseases and older age were associated with an increased risk of both incidence and poor COVID-19 clinical outcomes. For example, the United Kingdom and Italy analyzed COVID-19 cases and demonstrated that older age was associated with poorer outcomes [38, 39]. This again proved that increasing vaccination rates among the elderly and populations with underlying conditions was the key to containing COVID-19 and reducing the disease burden of morbidity and mortality. However, we found that women had a higher prevalence rate and incidence of severe clinical outcomes than men, which was contrary to the results of most other studies [40, 41]. Research in Canada had the same result as ours: Although the absolute number of COVID-19 cases was higher for females, they had a lower COVID-19 incidence rate after excluding the high-risk populations; more specifically, healthcare workers and long-term care residents, which are predominantly females [42]. Therefore, the true risk of sex during the Omicron outbreak needs to be further analyzed in depth in relation to age and occupation. Accordingly, we cannot draw any conclusions from the current data.

Interestingly, we found that smokers had a lower risk of both infection and severe clinical outcomes. The role played by smoking in COVID-19 infection has remained an open debate since the pandemic. Lippi G et al. published two articles in the European Journal of Internal Medicine to discuss this paradox. They summarized some large cohort studies or meta-analyses published on this topic in recent years [43–45] and considered that a simple conclusion that current smoking increases the risk of clinical deterioration in COVID-19 is largely unsupported according to the currently available scientific evidence [46, 47]. Simons et al. found that current smokers had a lower risk of SARS-CoV-2 infection than never smokers [43], and Chen et al. revealed that current smokers had an over 30% lower risk of COVID-19-related death (hazard ratio (HR), 0.57; 95%CI, 0.49–0.67) [44]. Both of the results were the same with us. In Thailand, Papadopoulos et al. conducted in-depth studies on the mechanisms of smoking pertaining to COVID-19 and concluded that environmental and novel genetic mechanisms may independently or jointly contribute to transient and serendipitous SARS-CoV-2 protection [48]. Nevertheless, the unfavorable consequences of cigarette smoking are paramount and indisputable, the effects are short-lived and gradually become irrelevant. Smoking is still the most potent common oxidant challenge encountered by the human respiratory tract that damages the arterial endothelium and wall, ultimately predisposing patients to a lethal COVID-19 disease course.Since the introduction of the Omicron variant worldwide, the effect of vaccination against the original strain on infection has attracted much attention [7, 49]. We found that, compared with the unvaccinated population, being vaccinated ≤ 3 times for COVID-19 had no significant effect on preventing infection but had a good effect on reducing the severity of clinical symptoms. It is generally recognized that the 3 dosages of vaccination have limited protective effect against Omicron. For example, Accorsi Ek et al. found that receipt of 3 doses of mRNA vaccine, relative to being unvaccinated and to receipt of 2 doses, was associated with protection against both the Omicron and Delta variants, although the higher odds ratios for Omicron suggest less protection for Omicron than for Delta [49]. A study in Hong Kong found that 100 days after receiving three or four doses of inactivated COVID-19 vaccine, the vaccine’s protective effect decayed to 6% and 11% of its original level, respectively [50]. However, Angel Paternina-Caicedo and her team found that non-mRNA vaccines showed little or no sustained protection against symptomatic COVID-19 during Omicron predominant periods in adult populations and the boosters of non-mRNA vaccines (i.e., CoronaVac, ChAdOx1, and Ad26.COV2. S) did not show protection against symptomatic Omicron during the entire follow-up period [51].

However, completion of the four doses of the COVID-19 vaccination significantly prevented infection and severe illnesses. It may result in that the four-dose COVID-19 vaccine strategy is a reinforced vaccine strategy specifically developed against the Omicron variant. The general population in China began to receive it on December 13 (i.e., during the epidemic period); therefore, better immune effects were expected.

### Limitations

However, this study had some limitations. First, this survey was conducted online, and participants were biased toward highly educated adults who were proficient in using the internet, with higher participation from females and lower coverage of infants, young children, and the elderly. The age structure of the surveyed population differed from that of Sichuan Province, leading to information bias. Second, although the total population was large, the information obtained was self-reported. Particularly, for cases defined based on clinical symptoms, the survey may have included a small number of individuals with fever and cough caused by other respiratory viruses, thereby reducing the reliability of the survey results. Third, owing to the lack of data on hospitalization and mortality cases during the outbreak, the disease burden of COVID-19 was inadequately seized. Fourth, for the evaluation of COVID-19 vaccine effectiveness, only the number of COVID-19 vaccine doses received by residents was surveyed, and detailed information on the type and timing of the COVID-19 vaccine received was not obtained. Therefore, extrapolation of our conclusions about vaccine effects should be cautious. Further research is warranted to identify appropriate correlates of protection for inactivated COVID-19 vaccines.

## Conclusions

The peak of the first wave of Omicron infection in Sichuan Province had passed and could be considered a snapshot of China under the new control strategy. Asymptomatic infections were rare in this large-scale outbreak and living in urban areas was associated with a higher risk of infection. Furthermore, there is a significant increase in the risk of severe clinical outcomes among females, those with chronic diseases, and the elderly. The vaccines have been effective in reducing poor clinical outcomes. What’s more, routine surveillance systems must be improved and optimized under the extreme conditions of highly infectious diseases.

## Data Availability

The data used in this article can be made available by the corresponding author upon request.

## Abbreviations

CDC: Centers for Disease Control and Prevention
OR: Odds ratio
CI: Confidence interval
COPD: Chronic obstructive pulmonary disease
AID: Autoimmune diseases

## References

[1] Organization World Health. COVID-19 weekly epidemiological update. 2023. https://www.who.int/publications/m/item/weekly-epidemiological-update-on-covid-19---22-march-2023. Accessed 22 Mar 2023.

[2] Hu J, Peng P, Cao X, Wu K, Chen J, Wang K (2022). Increased immune escape of the new SARS-CoV-2 variant of concern Omicron. Mol Immunol.

[3] Organization World Health, fourteenth meeting of the International Health Regulations. Statement on the (2005) Emergency Committee regarding the coronavirus disease (COVID-19) pandemic. 2023. https://www.who.int/news/item/30-01-2023-statement-on-the-fourteenth-meeting-of-the-international-health-regulations-(2005)-emergency-committee-regarding-the-coronavirus-disease-(covid-19)-pandemic. Accessed 12 Apr 2023.

[4] Viana R, Moyo S, Amoako DG, Tegally H, Scheepers C, Althaus CL (2022). Rapid epidemic expansion of the SARS-CoV-2 Omicron variant in southern Africa. Nature.

[5] Elliott P, Bodinier B, Eales O, Wang H, Haw D, Elliott J (2022). Rapid increase in Omicron infections in England during December 2021: REACT-1 study. Science.

[6] Elliott P, Eales O, Bodinier B, Tang D, Wang H, Jonnerby J (2022). Dynamics of a national Omicron SARS-CoV-2 epidemic during January 2022 in England. Nat Commun.

[7] Cerqueira-Silva T, De Araujo Oliveira V, Paixão Es F, Ptv P, Go, Pearce N (2022). Vaccination plus previous infection: protection during the omicron wave in Brazil. Lancet Infect Dis.

[8] Meyerowitz EA, Richterman A, Bogoch II, Low N, Cevik M (2021). Towards an accurate and systematic characterisation of persistently asymptomatic infection with SARS-CoV-2. Lancet Infect Dis.

[9] Iuliano AD, Brunkard JM, Boehmer TK, Peterson E, Adjei S, Binder AM (2022). Trends in disease severity and health care utilization during the early Omicron variant period compared with previous SARS-CoV-2 high transmission periods - United States, December 2020-January 2022. MMWR Morb Mortal Wkly Rep.

[10] Strum E, Casagrande Y, Newton K, Unger J (2022). Healthcare workers benefit from second dose of COVID-19 mRNA vaccine: effects of partial and full vaccination on sick leave duration and symptoms. Public Health Pract (Oxf).

[11] Regev-Yochay G, Amit S, Bergwerk M, Lipsitch M, Leshem E, Kahn R (2021). Decreased infectivity following BNT162b2 vaccination: a prospective cohort study in Israel. Lancet Reg Health Eur.

[12] Liu J, Liu M, Liang WJ (2022). The dynamic COVID-zero strategy in China. China CDC Wkly.

[13] Burki T (2023). Moving away from zero COVID in China. Lancet Respir Med.

[14] Wei Z, Ma W, Wang Z, Li J, Fu X, Chang H (2023). Household transmission of SARS-CoV-2 during the Omicron wave in Shanghai, China: a case-ascertained study. Influenza Other Respir Viruses.

[15] Xin H, Wang Z, Feng S, Sun Z, Yu L, Cowling B (2023). Transmission dynamics of SARS-CoV-2 Omicron variant infections in Hangzhou, Zhejiang, China, January-February 2022. Int J Infect Dis.

[16] Ruan F, Zhang X, Xiao S, Ni X, Yin X, Ye Z (2022). An outbreak of the SARS-CoV-2 omicron variant BA.1 - Zhuhai City, Guangdong Province, China, Jan 13, 2022. China CDC Wkly.

[17] Xinhua. China Focus: COVID-19 response further optimized with 10 new measures. 2022. http://english.news.cn/20221207/ca014c043bf24728b8dcbc0198565fdf/c.html. Accessed 4 Apr 2023.

[18] Boelle P, Obadia T. _R0: Estimation of R0 and Real-Time Reproduction Number from Epidemics_. R package version 1.2–10. 2022. https://CRAN.R-project.org/package=R0. Accessed 5 Mar 2023.

[19] Kolde R. _pheatmap: Pretty Heatmaps_. R package version 1.0.12. 2019. https://CRAN.R-project.org/package=pheatmap. Accessed 8 Mar 2023.

[20] Du J, Wang JM, Wang J, Gao Y, Pang XH, Li G (2022). Study of transmissibility of 2019-nCoV Omicron variant in Beijing. Chin J Epidemiol.

[21] Sun K, Tempia S, Kleynhans J, Von Gottberg A, Mcmorrow ML, Wolter N (2023). Rapidly shifting immunologic landscape and severity of SARS-CoV-2 in the Omicron era in South Africa. Nat Commun.

[22] Erikstrup C, Laksafoss Ad, Gladov J, Kaspersen Ka, Mikkelsen S, Hindhede L (2022). Seroprevalence and infection fatality rate of the SARS-CoV-2 Omicron variant in Denmark: a nationwide serosurveillance study. Lancet Reg Health Eur.

[23] Leung K, Lau EHY, Wong CKH, Leung GM, Wu JT (2023). Estimating the transmission dynamics of SARS-CoV-2 Omicron BF.7 in Beijing after adjustment of the zero-COVID policy in November-December 2022. Nat Med.

[24] Zheng L, Liu S, Lu F (2023). Impact of National Omicron outbreak at the end of 2022 on the future outlook of COVID-19 in China. Emerg Microbes Infec.

[25] Kim D, Ali St, Kim S, Jo J, Lim Js, Lee S, et al. Estimation of serial interval and reproduction number to quantify the transmissibility of SARS-CoV-2 omicron variant in South Korea. Viruses. 2022;14(3). 10.3390/v14030533. 10.3390/v14030533 PMC8948735 35336939

[26] Antonelli M, Penfold Rs, Merino J, Sudre C, Molteni E, Berry S (2022). Risk factors and disease profile of post-vaccination SARS-CoV-2 Infection in UK users of the COVID symptom study app: a prospective, community-based, nested, case-control study. Lancet Infect Dis.

[27] Silver A. Could rural China’s healthcare deal with covid-19? BMJ. 2021;375:n2759. 10.1136/bmj.n2759. 10.1136/bmj.n2759 34824132

[28] Chu Yr Z, Dl, Chen Y, Yi B, Lei S, Zhang Y (2022). Optimization of COVID-19 prevention and control measures based on prevalence characteristics of SARS-CoV-2 Delta and Omicron variants: an analysis on surveillance data of Ningbo city. Chin J Public Health.

[29] Commission Chinese National Health. Protocol of prevention and control for COVID-19 (7 edition). 2020. http://www.nhc.gov.cn/jkj/s3577/202009/318683cbfaee4191aee29cd774b19d8d.shtml. Accessed 3 Apr 2023.

[30] Ae R, Am P, Harding-Edgar L (2020). Covid-19 mass testing programmes. BMJ.

[31] Menni C, Valdes Am, Polidori L, Antonelli M, Penamakuri S, Nogal A (2022). Symptom prevalence, duration, and risk of hospital admission in individuals infected with SARS-CoV-2 during periods of omicron and delta variant dominance: a prospective observational study from the ZOE COVID study. Lancet.

[32] %J European Heart Journal (2022). Clinical presentation, disease course, and outcome of COVID-19 in hospitalized patients with and without pre-existing cardiac disease: a cohort study across 18 countries. Eur Heart J.

[33] Iacobucci G (2021). Covid-19: runny nose, headache, and fatigue are commonest symptoms of omicron, early data show. BMJ.

[34] Thompson MG, Yoon SK, Naleway AL, Meece J, Fabrizio TP, Caban-Martinez AJ (2022). Association of mRNA vaccination with clinical and virologic features of COVID-19 among US essential and frontline workers. JAMA.

[35] Taslem Mourosi J, Anwar S, Hosen MJ (2022). The sex and gender dimensions of COVID-19: a narrative review of the potential underlying factors. Infect Genet Evol.

[36] Luo H, Liu S, Wang Y, Phillips-Howard PA, Ju S, Yang Y (2020). Age differences in clinical features and outcomes in patients with COVID-19, Jiangsu, China: a retrospective, multicentre cohort study. BMJ open.

[37] Liu Y, Mao B, Liang S, Yang JW, Lu HW, Chai YH, et al. Association between age and clinical characteristics and outcomes of COVID-19. Eur Respir J. 2020;55(5). 10.1183/13993003.01112-2020. 10.1183/13993003.01112-2020 PMC7173682 32312864

[38] Iaccarino G, Grassi G, Borghi C, Ferri C, Salvetti M, Volpe M (2020). Age and multimorbidity predict death among COVID-19 patients: results of the SARS-RAS study of the Italian society of hypertension. Hypertension.

[39] Koh J, Shah SU, Chua PE, Gui H, Pang J (2020). Epidemiological and clinical characteristics of cases during the early phase of COVID-19 pandemic: a systematic review and meta-analysis. Front Med (Lausanne).

[40] Chen N, Zhou M, Dong X, Qu J, Gong F, Han Y (2020). Epidemiological and clinical characteristics of 99 cases of 2019 novel coronavirus pneumonia in Wuhan, China: a descriptive study. Lancet.

[41] Global Health 50/50. 2020. https://globalhealth5050.org/covid19/age-and-sexdata/. Accessed 4 Apr 2023.

[42] O'brien J, Du KY, Peng C (2020). Incidence, clinical features, and outcomes of COVID-19 in Canada: impact of sex and age. J Ovarian Res.

[43] Simons D, Shahab L, Brown J, Perski O (2021). The association of smoking status with SARS-CoV-2 infection, hospitalization and mortality from COVID-19: a living rapid evidence review with Bayesian meta-analyses (version 7). J Addiction.

[44] Chen UI, Xu H, Krause TM, Greenberg R, Dong X, Jiang X (2022). Factors associated with COVID-19 death in the United States: cohort study. JMIR Public Health Surveill.

[45] Tomaselli V, Ferrara P, Cantone GG, Romeo AC, Rust S, Saitta D, et al. The effect of laboratory-verified smoking on SARS-CoV-2 infection: results from the Troina sero-epidemiological survey. 2022;17(6):1617-30. 10.1007/s11739-022-02975-1 10.1007/s11739-022-02975-1 PMC9007731 35419722

[46] Lippi Giuseppe, Henry Brandon Michael (2020). Active smoking is not associated with severity of coronavirus disease 2019 (COVID-19). Eur J Intern Med.

[47] Lippi G, Henry BM, Sanchis-Gomar F (2023). COVID-19 and smoking: considerations after two years. Eu J Intern Med.

[48] Papadopoulos Ki, Papadopoulou A, Aw T. Live to die another day: novel insights may explain the pathophysiology behind smoker’s paradox in SARS-CoV-2 infection. Mol Cell Biochem. 2023:1–10. 10.1007/s11010-023-04681-8. 10.1007/s11010-023-04681-8 PMC9983545 36867341

[49] Accorsi EK, Britton A, Fleming-Dutra KE, Smith ZR, Shang N, Derado G (2022). Association between 3 doses of mRNA COVID-19 vaccine and symptomatic infection caused by the SARS-CoV-2 Omicron and Delta variants. JAMA.

[50] Lau JJ, Cheng SMS, Leung K, Lee CK, Hachim A, Tsang LCH (2023). Real-world COVID-19 vaccine effectiveness against the Omicron BA.2 variant in a SARS-CoV-2 infection-naive population. Nat Med.

[51] Paternina-Caicedo A, Quevedo DS, Ríos DS, Moyano D, Alvis-Guzmán N, Alviz-Zakzuk NR (2023). Comparative effectiveness and duration of protection of ChAdOx1, CoronaVac, BNT162b2, mRNA-1273, and Ad26.COV2.S COVID-19 vaccines for symptomatic and hospitalized Mu, Delta, and Omicron: a test-negative case-control study. Vaccine.

